# Scoring tools to identify TB patients facing catastrophic costs in the Philippines

**DOI:** 10.5588/pha.23.0014

**Published:** 2023-06-21

**Authors:** T. Yamanaka, A. M. C. Garfin, D. M. G. Gaviola, R. M. Arao, F. Morishita, T. Hiatt, N. Nishikiori, R. P. Yadav

**Affiliations:** 1 World Health Organization (WHO), Global Tuberculosis Programme, Geneva, Switzerland; 2 Department of Global Health and Development, London School of Hygiene & Tropical Medicine, London, UK; 3 School of Tropical Medicine and Global Health, Nagasaki University, Nagasaki, Japan; 4 National TB Control Programme, Department of Health, Manila, The Philippines; 5 Health Policy Development Programme (HPDP) UPecon Foundation, Inc., Quezon City, The Philippines; 6 WHO Regional Office for the Western Pacific, Manila, The Philippines; 7 WHO Country Office, Manila, The Philippines

**Keywords:** tuberculosis, TB costs, predictive value, risk assessment, The Philippines

## Abstract

**BACKGROUND:**

This study was to meet a practical need to design a simple tool to identify TB patients who may potentially be facing catastrophic costs while seeking TB care in the public sector. Such a tool may help prevent and address catastrophic costs among individual patients.

**METHODS:**

We used data from the national TB patient cost survey in the Philippines. We randomly allocated TB patients to either the derivation or validation sample. Using adjusted odds ratios (ORs) and β coefficients of logistic regression, we developed four scoring systems to identify TB patients who may be facing catastrophic costs from the derivation sample. We validated each scoring system in the validation sample.

**RESULTS:**

We identified a total of 12 factors as predictive indicators associated with catastrophic costs. Using all 12 factors, the β coefficients-based scoring system (area under the curve [AUC] 0.783, 95% CI 0.754–0.812) had a high validity. Even with seven selected factors with OR > 2.0, the validity remained in the acceptable range (β coefficients-based: AUC 0.767, 95% CI 0.737–0.798).

**CONCLUSION:**

The β coefficients-based scoring systems in this analysis can be used to identify those at high risk of facing catastrophic costs due to TB in the Philippines. Operational feasibility needs to be investigated further to implement this in routine TB surveillance.

## Abstract (fr)

**CONTEXTE:**

Cette étude visait à répondre à un besoin pratique de concevoir un outil simple pour identifier les patients atteints de TB qui pourraient potentiellement être confrontés à des coûts catastrophiques lorsqu'ils recherchent des soins de TB dans le secteur public. Un tel outil pourrait aider à prévenir et à traiter les coûts catastrophiques chez les patients individuels.

**MÉTHODES:**

Nous avons utilisé des données de l'enquête nationale sur les coûts des patients atteints de TB aux Philippines. Nous avons réparti aléatoirement les patients atteints de TB dans l'échantillon de dérivation ou de validation. À l'aide des odds ratio (OR) ajustés et des coefficients β de la régression logistique, nous avons développé quatre systèmes de notation pour identifier les patients atteints de TB qui pourraient être confrontés à des coûts catastrophiques à partir de l'échantillon de dérivation. Nous avons validé chaque système de notation dans l'échantillon de validation.

**RÉSULTATS:**

Nous avons identifié un total de 12 facteurs en tant qu'indicateurs prédictifs associés à des coûts catastrophiques. En utilisant les 12 facteurs, le système de notation basé sur les coefficients β (aire sous la courbe [AUC] 0,783 ; IC 95% 0,754–0,812) avait une validité élevée. Même avec sept facteurs sélectionnés avec OR > 2,0, la validité est restée dans la plage acceptable (basée sur les coefficients β : AUC 0,767 ; IC 95% 0,737–0,798).

**CONCLUSION:**

Les systèmes de notation basés sur les coefficients β dans cette analyse peuvent être utilisés pour identifier les personnes à haut risque de faire face à des coûts catastrophiques liés à la TB aux Philippines. La faisabilité opérationnelle doit être étudiée plus avant pour mettre en œuvre cela dans la surveillance de routine de la TB.

TB is a disease that affects socio-economically deprived individuals and is linked to poverty.^1,2^ Although public health facilities of many low- and middle-income countries provide TB care for free, TB patients and their households still suffer from a substantial financial burden for care-seeking, diagnosis and treatment.^3–5^ Hence, a target of achieving “zero catastrophic costs” for TB-affected households by 2030 was set in the WHO End TB Strategy.^6^

The WHO recommended that countries conduct a first national TB patient cost survey (TB-PCS) by 2020. The survey aims at establishing the baseline towards eliminating catastrophic costs due to TB. The WHO also suggested that governments repeat the surveys every 5 years to monitor progress.^6,7^ The Philippines is one of the 30 high TB burden countries and implemented the first TB-PCS in 2015–2016 as one of the pioneering countries.^8,9^ However, none of the countries has conducted the second TB-PCS, including the Philippines. Therefore, progress towards zero catastrophic costs after the first TB-PCS is unknown. This delay may be due to a lack of planning, as implementing a national survey requires substantial time and resources. Even if countries conduct the survey every 5 years as recommended by the WHO, it does not allow national TB programmes (NTPs) to monitor the situation on a more routine and frequent basis, nor identify areas or populations that need interventions to address or prevent catastrophic costs.

In response to such practical needs of the programme in the Philippines, this study aimed to develop a risk scoring system as a tool to identify TB patients who are currently or potentially at risk of incurring catastrophic costs. Therefore, the main objective of this study was to identify potential predictors associated with catastrophic costs due to TB using the TB-PCS data in the Philippines. Another aim was to develop and validate four risk scoring systems. The programme may use a risk scoring system as a proxy assessment tool for TB-PCS in routine TB services and surveillance to identify TB patients who may be facing catastrophic costs.

## METHOD

### Study population

This study was a secondary analysis using anonymised TB patient data collected during the Philippines’ TB-PCS. This study was conducted by the Philippines NTP and the University of the Philippines in 2016–2017.^10^ A total of 1,912 TB patients participated in the survey. The overall proportion of the households facing catastrophic costs was 42.4%.^8–10^ The sample size calculation and results are detailed elsewhere.^10^

### Statistical analyses

We used a split-sample comparison method to identify predictive factors, and develop and validate the scoring systems. Half of the total survey participants were randomly selected and allocated to a population group to develop the scoring systems (the derivation sample). The rest was assigned to another group to assess the validity of each scoring system (the validation sample).

We used descriptive statistics such as median and interquartile ranges (IQRs), mean and standard deviation (SD) for continuous variables, and frequencies and percentages for categorical variables to characterise the derivation and validation samples. Logistic regression was used to identify predictive factors and develop the scoring systems from the derivation sample. Statistical significance was defined at *P* < 0.05, and all of the statistical analyses and data visualisations were performed using R v4.0.3 (R Computing, Vienna, Austria).

This study did not consider the weight adjustment for drug-susceptible TB (DS-TB) and drug-resistant TB (DR-TB) because obtaining the nationally representative results was not the purpose of this study. Besides, we aimed to keep the analysis as simple as possible.

### Derivation of scoring systems

We first carried out univariate logistic regression analysis using the derivation sample to identify demographic, clinical and economic factors associated with households facing catastrophic costs due to TB, including coping mechanisms and perceived financial impact. We then performed a stepwise analysis with ‘forward selection’ to build the final multivariate model, in which factors with correlations or with a large *P*-value were removed.

Adjusted odds ratios (ORs) and β coefficients of multivariate logistic regression were used in this analysis to build scoring systems. First, we used categorical values derived from ORs based on a scoring algorithm. (Scoring System 1).^11,12^ In Scoring System 1, an integer score ranging from 0 to 6 based on OR was assigned to each predictive factor (Table 1: OR-based algorithm). We also created another scoring system using β coefficients of multiple logistic regression. (Scoring System 2: β coefficient-based algorithm).^13^ In Scoring System 2, β coefficients were multiplied by 10 and then rounded up to the nearest integer. A score of 0 was allocated for the reference category in each predictive factor in both scoring systems. Complete-case analysis was applied for handling missing data.

**TABLE 1:** OR- and β coefficient-based scoring algorithms

Scoring algorithm	Scoring
OR-based algorithm^11,12^	1.0 ≤ OR < 1.2: weight 1
	1.2 ≤ OR < 1.4: weight 2
	1.4 ≤ OR < 1.6: weight 3
	1.6 ≤ OR < 1.8: weight 4
	1.8 ≤ OR < 2.0: weight 5
	OR ≥ 2.0: weight 6
	Reference category: weight 0
β coefficient-based algorithm^13^	β coefficients were multiplied by 10 and then rounded to the nearest integer

OR = odds ratio.

We assessed the validity of both Scoring Systems 1 and 2 using only selected factors with a relatively high OR (OR > 2.0 in multivariate logistic regression) (short scale), as well as that with all the identified predictive factors (full scale). We did this to minimise operational workload for healthcare workers providing TB services.

### Validation of scoring systems

Total scores with each scoring system were calculated for each TB patient in the validation sample. We then assessed sensitivity, specificity and positive predictive value (PPV) at different thresholds (cut-off points) in each scoring system, and the optimal cut-off point was defined based on the point at which the Youden Index (sensitivity + specificity – 1) was maximised.^14^ Finally, the overall accuracy of each scoring system was evaluated based on the receiver-operating characteristics (ROC) curve and its area under the curve (AUC).^15^

### Ethics approval

Ethics clearance was not required as we used secondary data from the TB-PCS in the Philippines, which the Philippines NTP conducted in 2016–2017.^10^ As the data were already anonymised before conducting this study, we did not use any personal identifying information throughout the analysis.

## RESULTS

### Characteristics of samples

After random allocation of the total population (*n* = 1,912) to the derivation sample (*n* = 956) and validation sample (*n* = 956), the unweighted proportion of TB patients incurring catastrophic costs was 47.0% in the derivation sample and 45.1% in the validation sample (Table 2). In the derivation sample, 64.3% of the participants were male, with a mean age of 40 years (SD 19.7). In the validation sample, 65.6% of the participants were male, with a mean age of 40 years (SD 19.6). A large majority of the sample was new cases (73.3% in the derivation sample and 76.4% in the validation sample). The proportion of patients with DR-TB was 17.5% in the derivation sample and 16.0% in the validation sample. The mean monthly household income was US$233 in the derivation sample and US$260 before TB diagnosis, which fell to US$190 and US$223 during TB treatment, respectively. The unweighted mean total TB patient cost from the onset of TB symptoms until the end of TB treatment was US$1,031 in the derivation sample and US$896 in the validation sample.

**TABLE 2:** Demographic and clinical characteristics of derivation and validation samples from TB patient cost survey

Characteristic		Derivation sample		Validation sample		*P* value
		Total (*n* = 956) *n* (%)	With catastrophic costs (*n* = 449, 47%) *n* (%)	Total (*n* = 956) *n* (%)	With catastrophic costs (*n* = 431, 45.1%) *n* (%)	
Sex	Female	341 (35.7)	152 (33.9)	329 (34.4)	147 (34.1)	0.598
	Male	615 (64.3)	297 (66.1)	627 (65.6)	284 (65.9)	
Age, years, mean ± SD		40 ± 19.7	42 ± 17.9	40 ± 19.6	42 ± 18.7	0.921
Age group, years	0–14	96 (10.0)	29 (6.5)	84 (8.8)	26 (6.0)	0.668
	15–24	137 (14.3)	53 (11.8)	157 (16.4)	55 (12.8)	
	25–34	148 (15.5)	70 (15.6)	149 (15.6)	83 (19.3)	
	35–44	169 (17.7)	94 (20.9)	156 (16.3)	70 (16.2)	
	45–54	149 (15.6)	79 (17.6)	156 (16.3)	77 (17.9)	
	55–64	151 (15.8)	78 (17.4)	137 (14.3)	59 (13.7)	
	≥65	106 (11.1)	46 (10.2)	117 (12.2)	61 (14.2)	
Insurance status	No	380 (39.7)	165 (36.7)	406 (42.5)	175 (40.6)	0.245
	Yes	576 (60.3)	284 (63.3)	550 (57.5)	256 (59.4)	
Employment status before TB diagnosis	Employed	500 (52.3)	275 (61.2)	495 (51.8)	253 (58.7)	0.855
	Unemployed	456 (47.7)	174 (38.8)	461 (48.2)	178 (41.3)	
Patient was the main income earner	No	530 (55.4)	216 (48.1)	585 (61.2)	240 (55.7)	0.012
	Yes	426 (44.6)	233 (51.9)	371 (38.8)	191 (44.3)	
Household size	1–6	443 (46.3)	236 (52.6)	439 (45.9)	217 (50.3)	0.891
	≥7	513 (53.7)	213 (47.4)	517 (54.1)	214 (49.7)	
TB registration group	New	701 (73.3)	254 (56.6)	730 (76.4)	263 (61.0)	0.493
	Relapse	164 (17.2)	123 (27.4)	147 (15.4)	107 (24.8)	
	Retreatment (other than relapse)	87 (9.1)	69 (15.4)	76 (7.9)	58 (13.5)	
	Unknown	4 (0.4)	3 (0.7)	3 (0.3)	3 (0.7)	
Drug resistance status	DS-TB	789 (82.5)	301 (67.0)	803 (84.0)	292 (67.7)	0.426
	DR-TB	167 (17.5)	148 (33.0)	153 (16.0)	139 (32.3)	
Mode of TB diagnosis	Bacteriologically confirmed	494 (51.7)	280 (62.4)	516 (54.0)	274 (63.6)	0.336
	Clinically diagnosed	462 (48.3)	169 (37.6)	440 (46.0)	157 (36.4)	
Treatment phase at the time of interview	Intensive phase	172 (18.0)	108 (24.1)	199 (20.8)	108 (25.1)	0.133
	Continuation phase	784 (82.0)	341 (75.9)	757 (79.2)	323 (74.9)	
Mode of TB treatment	Self-administered	145 (15.2)	44 (9.9)	144 (15.1)	49 (11.4)	0.992
	With treatment partner	807 (84.8)	402 (90.1)	809 (84.9)	380 (88.6)	
Hospitalised at time of interview	No	939 (98.2)	433 (96.4)	936 (97.9)	414 (96.1)	0.740
	Yes	17 (1.8)	16 (3.6)	20 (2.1)	17 (3.9)	
Hospitalised during current phase	No	925 (96.8)	422 (94.0)	917 (95.9)	398 (92.3)	0.394
	Yes	31 (3.2)	27 (6.0)	39 (4.1)	33 (7.7)	
Total number of facility visits, *n*, mean ± SD		193 ± 162.7	256 ± 192.7	191 ± 163.0	258 ± 199.5	0.786
Monthly household income (before TB diagnosis), USD, mean ± SD		233 ± 272	218 ± 286	260 ± 357	212 ± 269	0.067
Monthly household income (during TB treatment), USD, mean ± SD		190 ± 224	115 ± 140	223 ± 342	122 ± 191	0.013
Total TB patient costs, USD, mean ± SD		1,031 ± 2,298	1,973 ± 3,086	896 ± 1,403	1,724 ± 1,733	0.121

SD = standard deviation; DS-TB = drug-susceptible TB; DR-TB = drug-resistant TB; USD = US dollar.

No significant difference was observed in demographic, clinical, economic factors between the two sample populations except for the proportion of patients who were household breadwinners (46.6% in the derivation sample and 38.8% in the validation sample, *P* = 0.012) and monthly household income during TB treatment (*P* = 0.013).

### Predictive values for facing catastrophic costs

The final multivariate regression model included 12 factors as predictive indicators to identify TB patients incurring catastrophic costs: DR-TB (OR 5.2, β 1.64, *P* < 0.001), monthly household income <US$160 (OR 1.9, β 0.62, *P* < 0.001), relapse or retreatment cases (OR 2.6, β 0.94, *P* < 0.001), household size <7 (OR 1.5, β 0.35, *P* = 0.026), patient was breadwinner (OR 3.1, β 1.13, *P* < 0.001), unemployed during TB treatment (OR 3.2, β 1.16, *P* < 0.001), taking TB treatment with treatment partner (OR 1.9, β 0.65, *P* = 0.005), ever hospitalised during TB treatment (OR 7.8, β 2.05, *P* < 0.001), taking loans (OR 1.4, β 0.30, *P* = 0.085), interrupted schooling (OR 2.3, β 0.83, *P* = 0.032), food insecurity (OR 1.5, β 0.38, *P* = 0.055), perceived financial impact (OR 2.2, β 0.78, *P* = 0.014 for very serious impact) (Table 3).

**TABLE 3:** Predictive factors of catastrophic costs and scoring systems developed using ORs and β coefficients from the derivation sample

Variables	Category	Logistic regression						
		Univariate		Multivariate			Scoring systems	
		OR (95%CI)	*P*-value	OR (95%CI)	*P*-value	β coefficient	1	2
Urban/rural	Urban	Reference		―		―	―	―
	Rural	2.0 (1.56–2.63)	<0.001	―	―	―	―	―
Drug resistance status*	DS-TB	Reference		Reference		―	0	0
	DR-TB	12.6 (7.86–21.43)	<0.001	5.2 (2.81–9.80)	<0.001	1.639	6	16
Monthly household income before TB, USD/month	>167.6	Reference		Reference		―	―	―
	≤167.6	1.8 (1.42–2.37)	<0.001	1.9 (1.36–2.56)	<0.001	0.621	5	6
TB registration group*	New Relapse and retreatment	Reference		Reference		―	0	0
		5.7 (4.14–7.99)	<0.001	2.6 (1.63–4.02)	<0.001	0.936	6	9
Mode of TB diagnosis	Bacteriologically confirmed	Reference		―		―	―	―
	Clinically diagnosed	0.4 (0.34–0.57)	<0.001	―	―	―	―	―
Household size	≥7	Reference		Reference		―	0	0
	0–6	1.6 (1.24–2.08)	<0.001	1.4 (1.04–1.95)	0.026	0.351	3	4
Patient was main income earner*	No	Reference		Reference		―	0	0
	Yes	1.8 (1.36–2.27)	<0.001	3.1 (2.15–4.49)	<0.001	1.128	6	11
Employment at interview*	Employed	Reference		Reference		―	0	0
	Unemployed	2.3 (1.76–3.09)	<0.001	3.2 (2.17–4.71)	<0.001	1.157	6	12
Mode of treatment	Self-administered	Reference		Reference		―	0	0
	Treatment partner	2.3 (1.57–3.36)	<0.001	1.9 (1.22–3.03)	0.005	0.647	5	6
Hospitalised during treatment*	No	Reference		Reference		―	0	0
	Yes	9.7 (3.79–32.60)	<0.001	7.8 (2.80–27.72)0	<0.001	2.050	6	21
Taking loans	No	Reference		Reference		―	0	0
	Yes	1.7 (1.29–2.24)	<0.001	1.4 (0.96–1.91)	0.085	0.300	2	3
Selling household assets	No	Reference		―		―	―	―
	Yes	2.2 (1.37–3.50)	0.001	―	―	―	―	―
Interrupted schooling*	No	Reference		Reference		―	0	0
	Yes	3.2 (1.72–6.13)	<0.001	2.3 (1.09–5.04)	0.032	0.833	6	8
Social exclusion	No	Reference		―		―	―	―
	Yes	1.9 (1.30–2.75)	0.001	―	―	―	―	―
Food insecurity	No	Reference		Reference		―	0	0
	Yes	1.7 (1.26–2.34)	0.001	1.5 (0.99–2.15)	0.055	0.378	3	4
Perceived impact*	No impact	Reference		Reference		―	0	0
	Little	2.0 (1.36–3.06)	0.001	1.7 (1.02–2.70)	0.042	0.507	4	5
	Moderate	3.0 (2.10–4.43)	<0.001	2.4 (1.51–3.72)	<0.001	0.859	6	9
	Serious	3.4 (2.12–5.50)	<0.001	1.7 (0.94–3.13)	0.078	0.536	4	5
	Very serious	5.6 (3.47–9.12)	<0.001	2.2 (1.17–4.08)	0.014	0.779	6	8
Total maximum score	Full scale						60	109
	Short scale						42	86

*Included in the short scale.

OR = odds ratio; CI = confidence interval; USD = US dollar.

Using all 12 factors mentioned above, the possible total score ranged from 0 to 60 in the full scale of Scoring System 1 and ranged from 0 to 109 in the full scale of Scoring System 2. For the short scale of scoring systems, seven factors with OR more than 2.0 were used. As a result, the possible total score in the short scale was from 0 to 42 in Scoring System 1 and from 0 to 86 in Scoring System 2, respectively (Table 3).

### Validation of scoring systems

The AUC demonstrated that Scoring System 2 derived from β coefficients had higher validity than Scoring System 1. Scoring System 1 yielded an AUC of 0.764 (95% CI 0.735–0.794) in full scale and 0.756 (95% CI 0.726–0.787) in the short scale, while Scoring System 2 had an AUC of 0.783 (95% CI 0.754–0.812) in full scale and 0.767 (95% CI 0.737–0.798) in the short scale, showing the acceptable level of validity even in short scales (Figure).

**FIGURE:**
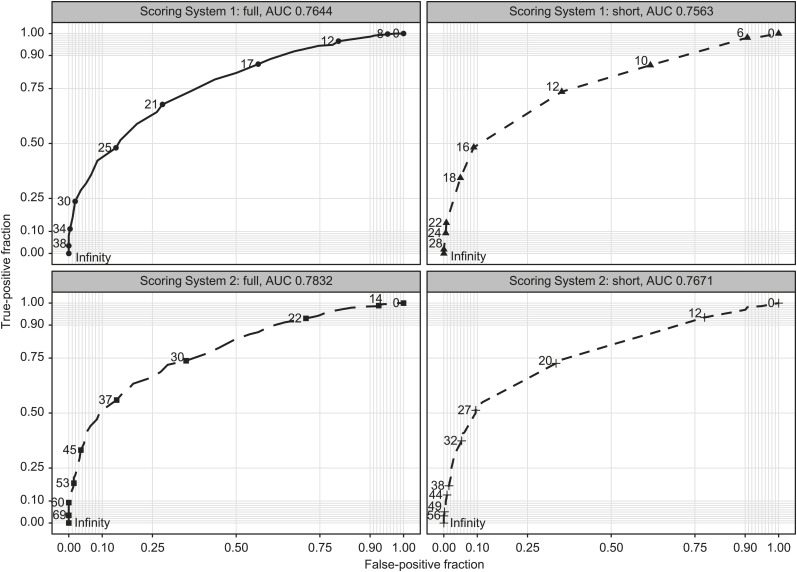
Receiver operating characteristic curves of the two scoring systems in the validation sample. AUC = area under the curve.

## DISCUSSION

In this study, we have developed risk scoring systems for predicting catastrophic costs due to TB using data from TB-PCS conducted in the Philippines. The survey had the largest sample size among all the TB patient cost surveys conducted to date. It allowed us to perform a derivation and validation process within the nationally representative sample of TB patients. A total of 12 sociodemographic and clinical characteristics were associated with facing catastrophic costs in the derivation sample.

We found that the β coefficients-based scoring systems had a high validity to predict the occurrence of catastrophic costs in the validation sample (with all 12 factors: AUC 0.783, 95% CI 0.754–0.812). In OR-based scoring systems, a score of 6 was given to all the risk factors that had OR of more than 2.0. This might result in ignoring the differences in the level of effect among the risk factors that were highly associated with facing catastrophic costs.^12^ For example, having both DR-TB as type of TB status and relapse/retreatment as the treatment group received the same score of 6, whereas the OR of DR-TB (OR 5.2) was two times higher than that of relapse/retreatment (OR 2.6). Also, in this study, scoring systems that used fewer risk factors showed an acceptable level of validity compared to the scoring systems with all 12 risk factors (with seven selected factors with a high OR: AUC 0.767, 95% CI 0.737–0.798). This implies that TB patients who faced catastrophic costs might share common risk factors with high ORs, such as having DR-TB, being hospitalised and being unemployed.

Given the global burden of TB disease, the End TB strategy has ambitious targets of ending the global TB epidemic by 2030. One of the targets is to achieve zero catastrophic costs for TB-affected households.^6^ A monitoring method for catastrophic costs has not been set yet since the target was newly set in 2013, and the implementation of TB-PCS was just started since 2015.^6,7^ This study is the first to develop a tool to be used in routine TB services/surveillance to identify patients with TB likely to face catastrophic costs.

The first TB-PCS in the Philippines revealed that 42.4% of TB-affected households incurred catastrophic costs.^10^ Although more than two-thirds of the households were already in poverty even before having TB, the proportion receiving a conditional cash transfer programme for the poor, the ‘*Pantawid Pamilyang Pilipino Program* (4Ps)’, was only 1.3% in the survey.^10^ This suggests that the 4Ps implemented by the Filipino Department of Social Welfare and Development (DSWD) may not have reached the TB-affected households in poverty despite their eligibility. A recent qualitative study conducted in South Africa found that even though TB patients were eligible for a disability grant, healthcare workers unintentionally or intentionally acted as gatekeepers to the grant.^16^ Also, the study in South Africa reported that the application process for the disability grant was time-consuming for TB patients and required extra costs, which resulted in limited access to the grant for TB patients.^16^ A similar issue was reported for a national disability grant for formal employers in TB-PCS in Lao People’s Democratic Republic,^17^ which found that a lengthy administrative process to access the grant might be a barrier for the low proportion of TB patients receiving the grant.^17^ It is necessary to better understand the reasons for the low proportion of 4Ps recipients among TB patients in the Filipino context. In any case, strengthening multisectoral cooperation/coordination between NTP and DSWD would certainly save TB-affected households from financial catastrophe.

A scoring system in our study (Scoring System 1: short scale) will be implemented as a questionnaire in an existing mobile application (‘Care TB’) in the Philippines.^18^ Health workers can create scores based on interviews with patients, or patients can self-assess and provide the necessary information to create the scores. These scores can serve as a proxy indicator for the patients facing catastrophic costs and alert the health worker for those at risk while suggesting possible interventions. The scores will also feed into an algorithm for further refining the accuracy of predictions of patients who will experience catastrophic costs. The trends produced by these assessments and resulting scores will be monitored regularly by the NTP as a part of TB surveillance. Findings will periodically be shared with the multisectoral National Coordinating Committee for TB, including the DSWD, to advocate for enhanced social protection for TB patients.

Our analysis had two limitations. First, identified risk factors and the scoring systems developed are specifically for Filipino settings and those who receive TB treatment in the NTP-engaged facilities. The proportion of catastrophic costs and their risk factors vary widely depending on the country context. Our scoring systems may therefore be specific to patients in the Philippines who undergo TB treatment in non-NTP-engaged facilities; however, the analytical methods used in this study can be applied to other countries.^8,19^ For example, while data on HIV status were routinely collected in other TB patient cost surveys, it was not collected in the Philippines TB-PCS due to the confidentiality of HIV status in the Philippines. Patients with TB-HIV coinfection usually incur a heavier financial burden due to comorbidity and the greater frequency of facility visits.^17,20^ In such contexts, HIV status may need to be a component of scoring systems. Second, It is important to note that while risk prediction models are valuable tools, they should be applied to populations or groups with available sociodemographic and clinical data rather than being used to assess individual patients.^21^ Hence, the scores obtained from our TB scoring system should not be used as a sole indicator to classify each TB patient with regards to catastrophic costs. Rather, it should be used in conjunction with other clinical assessments and considerations.

## CONCLUSIONS

The scoring systems developed in this analysis, which are based on β coefficients, can be valuable in identifying individuals who may potentially face catastrophic costs due to TB in the Philippines. These systems may serve as a practical tool to monitor progress towards the goal of zero catastrophic costs. Further investigation is required to assess the operational feasibility of implementing and using this as a tool in routine TB surveillance systems.
